# Bleeding and Thrombosis: Insights into Pathophysiology of *Bothrops* Venom-Related Hemostasis Disorders

**DOI:** 10.3390/ijms22179643

**Published:** 2021-09-06

**Authors:** Sébastien Larréché, Jean-Philippe Chippaux, Lucie Chevillard, Simon Mathé, Dabor Résière, Virginie Siguret, Bruno Mégarbane

**Affiliations:** 1 INSERM, UMRS-1144, Paris University, 75006 Paris, France; slarreche@hotmail.fr (S.L.); luciechevillard@gmail.com (L.C.); sim.mathe@gmail.com (S.M.); 2 Department of Medical Biology, Bégin Military Teaching Hospital, 94160 Saint-Mandé, France; 3 MERIT, IRD, Paris University, 75006 Paris, France; jean-philippe.chippaux@ird.fr; 4 CRT, Pasteur Institute, 75015 Paris, France; 5 Clinical Toxicology Unit, Critical Care Department, University Hospital of Martinique, Fort de France, 97200 Martinique, France; Dabor.RESIERE@chu-martinique.fr; 6 INSERM, UMRS-1140, Paris University, 75006 Paris, France; virginie.siguret@aphp.fr; 7 Laboratory of Hematology, Lariboisière Hospital, 75010 Paris, France; 8 Department of Medical and Toxicological Critical Care, Lariboisière Hospital, 75010 Paris, France

**Keywords:** snake venom, hemorrhage, microthrombi, thrombocytopenia, coagulopathy

## Abstract

Toxins from *Bothrops* venoms targeting hemostasis are responsible for a broad range of clinical and biological syndromes including local and systemic bleeding, incoagulability, thrombotic microangiopathy and macrothrombosis. Beyond hemostais disorders, toxins are also involved in the pathogenesis of edema and in most complications such as hypovolemia, cardiovascular collapse, acute kidney injury, myonecrosis, compartmental syndrome and superinfection. These toxins can be classified as enzymatic proteins (snake venom metalloproteinases, snake venom serine proteases, phospholipases A_2_ and L-amino acid oxidases) and non-enzymatic proteins (desintegrins and C-type lectin proteins). Bleeding is due to a multifocal toxicity targeting vessels, platelets and coagulation factors. Vessel damage due to the degradation of basement membrane and the subsequent disruption of endothelial cell integrity under hydrostatic pressure and tangential shear stress is primarily responsible for bleeding. Hemorrhage is promoted by thrombocytopenia, platelet hypoaggregation, consumption coagulopathy and fibrin(ogen)olysis. Onset of thrombotic microangiopathy is probably due to the switch of endothelium to a prothrombotic phenotype with overexpression of tissue factor and other pro-aggregating biomarkers in association with activation of platelets and coagulation. Thrombosis involving large-caliber vessels in *B. lanceolatus* envenomation remains a unique entity, which exact pathophysiology remains poorly understood.

## 1. Introduction

*Bothrops* snakes, also called lanceheads, are the main genus of medical importance in the Neotropical Americas. They are involved in most cases of envenomation in studies performed in Brazil [1,2,3,4,5], Ecuador [6], French Guiana [7], Colombia [8], Argentina [9], Costa Rica [10] and Panama [11]. Belonging to the Viperidae family and the Crotalinae subfamily, *Bothrops* genus comprises nearly 50 species, widely distributed in South and Central America (from southern Mexico to Argentina) and in the Caribbean. Lanceheads usually occupy terrestrial, arboreal and semiarboreal biotopes, but may be occasionally found in disturbed habitats around human settlements and urban areas [12,13]. The main species involved in human envenomations are *B. atrox* in the Amazon region, *B. asper* from southern Mexico to western Ecuador and Colombia, and *B. jararaca* in South East Brazil, Argentina and Paraguay, with a large body of medical literature for these three taxons [11,13,14,15,16]. *B. atrox* and *B. asper* are phylogenetically close and belong to the *B. atrox* group [12,17]. Fenwick et al. has proposed to assign *B. jararaca* to a new genus, *Bothropoides* [18], subsequently considered synonym of *Bothrops* [19].

Snake venoms contain a highly potent mixture of bioactive molecules, known as toxins. Toxins from *Bothrops* venom exhibit a great diversity and are predominately represented by snake venom metalloproteinases (SVMP), snake venom serine proteinases (SVSP), phospholipases A_2_ (PLA_2_), C-type lectin-like toxins (CTL), desintegrins, cysteine-rich secretory proteins (CRISP), L-amino acid oxidases (LAAO), natriuretic peptides including vasoactive peptides, bradykinin potentiating and inhibitory peptides [20]. *Bothrops* venoms have various targets against hemostasis. This process aims to stop or limit bleeding after vascular injury. The three distinct phases, primary hemostasis (mainly involving platelets), coagulation (related to fibrin formation) and fibrinolysis, are closely linked [21]. The von Willebrand factor (vWF) is a multimeric protein present in the subendothelium and in plasma where it is conformationally activated by shear forces. Injured vessel wall recruits platelets via vWF; platelets are activated, recruit additional platelets and form a platelet aggregate [22]. Initiation of the coagulation pathway is secondary to exposure to tissue factor (TF) and formation of TF/factor VII activated (VIIa) complex, which activates small amounts of FIX and FX. Then, two overlapping stages occur with amplification in which platelets and cofactors are activated to set the stage for large scale thrombin generation, and propagation in which large amounts of thrombin are generated on the platelet surface [23]. During fibrin formation, the N-terminal part of the Aα and Bβ chains (within the E region) are cleaved by thrombin, releasing fibrinopeptides A and B. This cleavage results in the unmasking of four binding sites on the E region, each site being able to bind to the C-terminal portion of a D region from fibrin monomers. In this way, monomeric fibrin self-assemblies spontaneously yield fibrin oligomers that lengthen to make two-stranded protofibrils [24]. Thrombin also activates factor XIII that cross-links fibrin polymers leading to the formation of an insoluble fibrin net. Several control mechanisms exist for localizing fibrin formation to the site of injury including tissue factor pathway inhibitor, protein C system, antithrombin, and glycosaminoglycans on the endothelium [25]. Fibrinolysis is a highly regulated process, starting with fibrin formation and activation of the tissue plasminogen activator (tPA) on plasminogen-binding sites. Release of tPA from endothelial cells leads to the conversion of proenzyme plasminogen into plasmin [26]. Plasmin digests the fibrin component of the blood clot. Inhibition of the fibrinolytic system occurs at the level of the plasminogen activator (by plasminogen activator inhibitor type 1, PAI-1) or at the level of plasmin (by α_2_-antiplasmin, α_2_-AP) [22]. 

Local features of *Bothrops* envenomation are pain, edema, bruising, bleeding from fang punctures, sometimes blistering or dermonecrosis [13,16,27]. Hemorrhagic syndrome remains the most important cause of lethality associated with these species [5,28,29], while other complications may occur: acute kidney injury (AKI), shock, myonecrosis, compartment syndrome and secondary infections [16,30]. 

*B. lanceolatus* and *B. caribbaeus*, found only in Martinique and Saint Lucia, respectively, also belong to the *B. atrox* group [12,17]. Despite an initial local presentation similar to that described during envenomation by *B. atrox* or *B. asper* including local hemorrhage and ecchymosis, envenomations due to these Caribbean species are unusually associated with systemic bleeding and incoagulabiblity, but may by contrast be complicated by multiple systemic infarctions within 48 h after the bite [31]. 

Thus, hemostasis disorders induced by *Bothrops* venoms are responsible, directly or indirectly, for a large number of clinical impairments, sources of marked morbidities and potential fatality. Understanding the underlying mechanisms is therefore challenging as prerequisite to optimize current management strategies and develop new approaches.

## 2. Clinical Manifestations Induced by Hemostasis Disorders during *Bothrops* Envenomations

### 2.1. Bleeding

#### 2.1.1. Local Hemorrhage

A discrete trickle of blood from the fang punctures starts minutes after the bite with venom injection, even in the absence of systemic signs and blood incoagulability [28,29,32,33]. A local ecchymosis around the bite site is another classical feature and seems more frequent in children [28,33]. Blisters around or far from the bite in the affected limb may appear within the first 24 h and contain a serohematic or a bloody content, to be considered as manifestation of local hemorrhagic syndrome [13].

#### 2.1.2. Systemic Hemorrhage

Mucosal hemorrhages are the earliest manifestations of systemic bleeding. Gingival bleeding and hematuria are the most frequent but other mucous membranes may occur with epistaxis, conjunctival bleeding, otorrhagia and metrorrhagia [28,29,32,33]. Hemorrhagic skin and subcutaneous manifestations are also reported including petechiae, ecchymosis or hematoma, bleeding through recent wounds or veni-puncture site [16,28,29,34].

Visceral hemorrhages are less frequent but more severe and may include hemarthrosis (finger joints, knee) [16], hepatic or retroperitoneal hematoma [16,35], hemothorax [32,36], pulmonary hemorrhage [37], hematemesis and rectal bleeding [28,29]. Central nervous system hemorrhages are a dreadful complication and may be intracerebral, intraventricular, subarachnoid, subdural, extradural, cerebellar or medullar [16,38,39]. In a study on the prevalence of cerebrovascular complications in *Bothrops* envenoming, it was found that 2.6% of the victims developed a cerebrovascular event, of which about 60% died and 40% remained with sequelae [40]. Delay in antivenom administration may contribute to the onset of such complications [16,38,41]. Systemic bleeding is significantly associated with higher snakebite fatality [5] and amputation [42].

#### 2.1.3. Thrombocytopenia and Incoagulability

Thrombocytopenia and unclottable blood on admission are independently associated with systemic bleeding during hospitalization for *B. atrox* et *B. jararaca* envenomation [29,43,44].

Clinical studies in patients bitten by *B. jararaca* evidenced that systemic bleeding is more frequent in patients with thrombocytopenia than in patients with blood incoagulability [45,46]. By contrast, thrombocytopenia is an infrequent event in envenomations in the Amazon, mainly caused by *B. atrox* [28,43]. A negative correlation was found between the number of platelets and the mean platelet volume on patient admission for an envenomation by *B. atrox*, suggesting peripheral platelet destruction, which tends to increase the mean platelet volume [29]. Thrombocytopenia on admission is also a useful prognostic indicator of local complication development such as necrosis [44]. 

The hemorrhagic syndrome is exacerbated by incoagulability [28,29]. Unclottable blood due to consumption coagulopathy and defibrinogenation usually occurs within one hour [16]. In contrast, patients with only local bleeding and without bleeding may often show normal levels of hemostatic factors and fibrinolysis parameters [43].

Envenomed patients present increased prothrombin time (PT) and activated partial thromboplastin time (aPTT), with low level of fibrinogen. Factors II, V, VII, X, XIII also decrease [43,47,48]. Factors involved in the intrinsic pathway (factors VIII, IX, XI, XII) are less frequently reduced [47,48]. 

PT and aPTT take respectively 3 and 1 day(s) to normalize without antivenom. Fibrinogen level reaches 100 mg/dL in 24 h [49]. In mice, injection of *B. jararaca* venom upregulates hepatic mRNA synthesis of fibrinogen chains, which may explain the fast recovery of clotting tests and fibrinogen level, even without antivenom [50].

Owing to its low cost, simplicity and good correlation with plasma fibrinogen concentration, the 20 min whole blood clotting test (20WBCT) is an effective and reliable method for evaluation of hemostatic status in the primary care centers without laboratory [51]. Increased levels of plasma TF are detected and may contribute to the intravascular generation of thrombin as suggested by high levels of thrombin-antithrombin complexes, leading to a transient hypercoagulable state, then to incoagulability and systemic bleeding [43,45,52]. 

Secondary hyperfibrinolysis was observed in patients envenomed by *B. atrox* in Manaus, especially those with systemic bleedings, high levels of D-dimers and fibrin/fibrinogen degradation products (FDP) and low levels of plasminogen and α_2_-AP [28,43]. This fibrinolysis seems to be in response to fibrin deposition, in order to avoid microvascular thrombotic obstruction.

High levels of PAI-1 and tPA as well as increase in vWF and thrombomodulin were recorded in patients bitten by *B. jararaca* evolving with systemic bleeding with or without incoagulable blood, suggesting that the fibrinolysis is also due to vascular endothelial damage [45,52].

### 2.2. Thrombosis

#### 2.2.1. Thrombotic Microangiopathy

Thrombotic microangiopathy (TMA) was recently described in envenomation cases by *B. jararaca* [53,54], *B. venezuelensis* [55] and *B. erythromelas* [56]. These patients presented on admission a classic local syndrome with pain, edema, local ecchymosis or bleeding at the fang punctures, but no systemic bleeding. Coagulopathy resolved within 12–24 h after antivenom infusion, whereas the features associated with TMA (thrombocytopenia, hemolytic anemia with presence of schistocytes in blood smears, decreased haptoglobin level, increased lacticodehydrogenase (LDH) level and AKI) tended to start 1–3 days post-bite despite antivenom and persisted for longer. In the case of *B. venezuelensis* bite, hyperfibrinogenemia began at day 2 and persisted over 2 weeks despite the administration of antivenom 4.5 h after the bite [55]. When assayed, ADAMTS13 activity and C3/C4 complement levels were within the normal range [54].

In a fatal case due to massive pulmonary hemorrhage occurring 1 h after *B. jararacussu* bite, autopsy reported evidence of multiple fibrin and platelet microthrombi in the subcutaneous and muscular tissue around the bite site, and in the heart and lung microcirculations [37]. Bleeding and microthrombosis may occur simultaneously and therefore be considered as part of the same nosological framework.

#### 2.2.2. Macrothrombosis

*B. lanceolatus* is responsible for 20–30 snakebite cases in Martinique each year [57]. Approximatively 30% to 40% of the *B. lanceolatus* envenomations are associated with systemic multifocal thrombotic complications, usually occurring within 5 h to a week after the bite, even after a moderate envenomation limited to local signs [31,58,59]. Thromboses involve cerebral, myocardial, pulmonary and femoral arteries, and may lead to death or major functional sequelae in the absence of specific antivenom [58]. More than half of the patients envenomed by *B. lanceolatus* have thrombocytopenia while rare patients develop disseminated intravascular coagulation. Thrombocytopenia, minimally reduced prothrombin, normal activated partial thromboplastin time, and elevated fibrinogen concentration are typical features in *B. lanceolatus*-envenomed patients who will further develop thromboses [57,59]. 

A fatal case of *B. lanceolatus* envenomation with diffuse thrombotic microangiopathy causing multiple cerebral, myocardial and mesenteric infarctions at autopsy was reported. Laboratory features associated hemolytic anemia with increased LDH and biblirubin levels, thrombocytopenia and hyperfibrinogenemia with minimally reduced prothrombin ratio and normal activated partial thromboplastin time [60].

A similar clinical presentation was reported in a patient bitten by *B. caribbaeus* in Saint-Lucia, who developed multiple cerebral infarctions [61]. 

### 2.3. Complications Associated to Hemostasis Disorders

Beyond the hemorrhagic consequences, microcirculation impairment around the bite site may partly explain the edematogenic process due to vascular permeability increase [62,63]. Additionally, systemic bleeding and hemostatic disturbances contribute to hypovolemia, hypotension, tissue hypoperfusion, cardiovascular shock and cerebrovascular events [5,64,65]. 

Presence of thrombi in arterioles and arteries associated to local microcirculation impairment may contribute to limit vascularization of distal muscle tissues leading to poor regeneration of muscles and local myonecrosis followed by secondary infection [27]. AKI is the leading cause of lethality among patients surviving the early effects of *Bothrops* venom [66]. Glomerular microthrombi due to venom-induced thrombin generation, endothelial injury and intravascular hemolysis represent the main pathways for *Bothrops* envenomation-related AKI development [67,68]. Finally toxins involved in thrombi may lead to systemic inflammation and amplify this process [69].

### 2.4. Response to Antivenom

Antivenom is the first-line treatment of snakebite envenomation and its related hemostasis disorders. It consists of polyclonal antibodies generated by immunizing animals with small amounts of snake venom. The resulting antibodies are purified from serum or plasma and formulated into intact IgG or F(ab’)_2_ or Fab-fragment therapies to be administered intravenously [70]. 

The majority of patients showed rapid restoration of blood coagulability and cessation of bleeding [32,34,71,72,73]. Bleedings (except hematuria) are corrected in most patients 6–12 h after antivenom following *B. atrox* [28,74] and *B. asper* [32,71] bites. Hematuria correction is slower, about 12–24 h after antivenom administration [16]. In some cases, patients without systemic bleeding on admission started to bleed after antivenom but these manifestations did not persist [28]. Blood coagulation is restored most of the time within 6–24 h after antivenom [28,32,48,71,72]. Clotting tests and fibrinolysis parameters return to normal around 48 h after antivenom [28,47,49].

In French Guiana, a non-local antivenom (Antivipmyn Tri^®^, Instituto Bioclon, Ciudad de México, Mexico) is currently used to treat snakebites including *B. atrox* envenomations. A retrospective study shown that compared to patients receiving no antivenom, the 3-vials dosage initially used had no benefit in correction of snakebite-related coagulopathy [49]. In a second study with an increased dosage to 6 vials for most patients, a reduction in the time to return to normal clotting tests was observed for patients who received this antivenom in comparison to those who did not, but with a higher rate of early adverse reactions [47]. It therefore appears necessary to optimize treatment and evaluate other antivenoms in this French overseas department.

*Bothrops* bite-related TMA occur despite specific antivenom but still have a good prognosis [53,55,56,60].

A monovalent antivenom against *B. lanceolatus* venom, Bothrofav^®^ (Micropharm Limited, Newcastle Emlyn, UK), prevents thrombosis if it is early given, up to 6 h following the bite [75]. Since its introduction in 1993, the mortality rate decreased [57]. However some patients developed cerebral infarctions within 24 h despite early administration of Bothrofav^®^ [76]. In these cases, as reported in cases of snakebite-related TMA, coagulopathy was quickly resolved whereas thromboses occurred with a delay [76]. Anticoagulant and thrombolytic treatment is of limited efficacy to prevent these thrombotic complications [31,58].

## 3. Toxins Involved in *Bothrops* Venom-Related Hemostasis Disorders

Snake venoms are complex mixtures of proteins with toxic activities, with many distinct isoforms, affecting different physiological targets, including enzymes and non-enzymatic proteins. Toxins isolated from *Bothrops* venoms and involved in hemostasis disorders are classified into different families.

### 3.1. Enzymatic Proteins

#### 3.1.1. Snake Venom Metalloproteinases

SVMP are zinc-dependent endopeptidases belonging to the metzincin family [77]. They represent the predominant protein family in *Viperinae* and *Crotalinae* venoms, especially in *Bothrops* venoms [20]. They display several activities resulting in hemorrhage, proteolytic degradation of fibrinogen and fibrin, activation of factors II (prothrombin) and X, apoptosis, platelet aggregation or inhibition, inflammation induction and inhibition of blood serine proteinase inhibitors [78]. Based on their size and domain structure, these multi-domain proteins have been classified in P-I, P-II and P-III classes [78].

The mature protein forms of P-I SVMP (20–30 kDa) contain a catalytic domain characterized by a zinc-binding sequence (HEXXHXXGXXH) followed by a conserved “Methionine-turn” motif. The P-I SVMP are divided into two subgroups, i.e., P-IA which induce hemorrhage and P-IB with weak (or no) hemorrhagic effects [79]. Structural comparisons of hemorrhagic and non-hemorrhagic P-I SVMP from snake venoms revealed differences in the loop comprising residues 153 to 176 adjacent to the methionine-turn, conferring a higher flexibility in the loop area of hemorrhagic P-I SVMP [80]. This difference could influence the interaction with relevant substrates in the extracellular matrix [81,82].

Class P-II includes enzymes containing the metalloproteinase domain described above and a disintegrin domain (30–60 kDa). Disintegrins have most often an RGD motif which is the primary recognition site for the integrin α_IIB_β_3_ (fibrinogen receptor, also known as GP IIb/IIIa) inhibiting platelet aggregation [83]. This canonical RGD motif is not always present in all P-II SVMP and could be replaced by a KGD, VGD, WGD, MLD, RTS or KTS motif [84]. 

P-III SVMP (60–100 kDa) contain a metalloproteinase catalytic domain and disintegrin-like and cysteine-rich domains in a single polypeptide chain, many of them being glycosylated. Proteolytic cleavage, repeated domain loss and presence of other ancillary domains are responsible for structural diversities of P-II and P-III SVMP. The former P-IV class characterized by the addition of a lectin-like domain is now considered as a P-III class (P-IIId) due to a post-translational modification [84]. Jararhagin is a hemorrhagic P-III SVMP, isolated from the venom of *B. jararaca* [85]. Because both recombinant jararhagin and native deglycosylated jararhagin did not show any hemorrhagic activity in a murine model, the hemorrhagic properties of SVMP seem to be dependent on post-translational modifications [86].

#### 3.1.2. Snake Venom Serine Proteases

SVSP have a typical trypsin fold with a highly reactive serine residue in their active site and catalyze the cleavage of specific covalent bonds in peptides and proteins [87]. These enzymes affect various systems including hemostasis, complement, blood pressure and nervous system. Activities of SVSP interfering with hemostasis may be procoagulant or anticoagulant. Procoagulant proteases are distinguished between enzymes able to activate factors II, VII and/or X and enzymes named thrombine-like enzymes (TLE) which cleave fibrinogen in fibrin. Other activities against hemostasis are direct fibrin(ogen)olysis, activation of protein C and plasminogen, inhibition of antithrombin, PAI-1 and α_2_-AP. SVSP isolated from *Bothrops* venoms are most often TLE or fibrin(ogen)olytic [87,88].

#### 3.1.3. Phospholipases A_2_

PLA_2_ are small proteins with the molecular mass of 13–15 kDa. PLA_2_ hydrolyze the ester linkages in glycerophospholipids at the *sn*-2 positions to produce equimolar amounts of lysophospholipids and free fatty acids. It requires calcium for their catalytic actions. Their structure has three major α-helices and two antiparallel β-sheets cross-linked by disulfides bonds. Snake venom PLA_2_ are classified into two groups, I and II, according to the location of disulfide bonds [89,90]. PLA_2_ from *Bothrops* venoms belong to group II. Asp_49_ and Lys_49_ are the most well studied PLA_2_ of group II. These PLA_2_ are classified according to the amino acid located at position 49 [91]. 

They display a wide range of biological effects such as neurotoxicity, cardiotoxicity, local or systemic myotoxicity, hemolysis, and anticoagulating, antiplatelet and edema-forming activities.

#### 3.1.4. L-Amino Acid Oxidases

LAAO are homodimeric flavoenzymes that catalyze the stereospecific oxidative deamination of L-amino acid to α-keto acid and produce hydrogen peroxide. These enzymes have a molecular mass of 110–159 kDa and three major domains: substrate-binding domain, FAD-binding domain and helical domain.

LAAO can inhibit or induce platelet aggregation, depending on the isoform. In addition, LAAO may be responsible for edema, inflammation, apoptosis, hemolysis and hemorrhage [92].

### 3.2. Non-Enzymatic Proteins

#### 3.2.1. Disintegrins

Disintegrins are small polypeptides (40–100 amino-acid) derived by proteolytic processing from P-II SVMP or from genes solely encoding disintegrins (so called true or short-coding disintegrins) [83]. They are carrying the integrin recognition motifs (RGD and its variants). They are divided into several groups, depending of their molecular mass and their number of cysteine bridges [93]. 

Most of their toxic effects are due to their interaction with cell surface ligands: inhibition of platelet aggregation, apoptosis, cytotoxicity. Moreover, they interfere with the functions of integrins by altering different cellular processes such as migration, adhesion or proliferation [94]. 

#### 3.2.2. C-Type Lectin Proteins

Snake venom CTL bind to mono- and oligo-saccharides in a calcium-dependent manner. They are distinguished between classical CTL possessing a carbohydrate recognition domain that binds to sugars, and non-sugar-binding C-type lectin-related proteins, also called snaclecs. Snaclecs are more widely present in snake venoms than classical CTL. While CTL are disulfide-linked homodimers or homo-oligomers, snaclecs are disulfide-bonded heterodimers or oligomeric complexes of heterodimers including two highly homologous subunits tightly associated by loop-swapping, with a concave surface between the two subunits. The concave surface is probably the main ligand binding site [95]. 

Most classical CTL isolated in snake venoms bind to galactose and induce platelet aggregation. Snaclecs could be agonists or antagonists of platelet aggregation, or anticoagulant (FIX-, FX- or α-thrombin-binding) [88,96].

### 3.3. Venom Variability

Venoms of *B. lanceolatus* and *B. caribbaeus*, rather associated with the occurrence of thrombosis, mainly contain SVMP, PLA_2_, SVSP, LAAO [97] and do not differ significantly in the composition from other venoms belonging to this genus responsible for systemic bleeding (Table 1). The total relative amount of SVMP was found to be approximatively the same in the venoms of *B. jararaca* and *B. lanceolatus*, the difference being in the distribution of SVMP subgroups. *B. lanceolatus* has relatively more P-I SVMP identified than *B. jararaca* venom while *B. jararaca* venom has a higher amount of hemorrhagic P-III SVMP [98]. On the other hand, Gutierrez et al. found a predominance of P-III SVMP in both *B. lanceolatus* and *B. caribbaeus* venoms [97].

These contradictory results could be explained by an important intraspecific variability linked to different factors such as sex, geographical distribution, ontogeny and captivity.

Sex-based variation of *B. atrox* venom was observed even in siblings. Male venom showed higher LAAO, PLA_2_ and hemorrhagic activities, while female venoms showed higher coagulant activity [103]. Venom from *B. jararacussu* males is more procoagulant than those of females [110].

A geographic and altitudinal venom variability was observed in *B. asper* lineages from North-Western South America, especially in the four major toxin families: SVMP (mainly PI- and PII-SVMP), PLA_2_, SVSP and CTL [105]. In the same way, *Bothrops venezuelensis* venoms, from five localities in the North-Central Venezuelan regions, showed differences in minimal hemorrhagic dose and fibrinolytic activity [111]. 

A linear relation between ontogenetic variability and snake size has been described in the venom composition of *B. atrox*, *B. asper* [112] and *B. jararacussu* [99]. Concentration of P-I SVMP is higher than that of P-III SVMP in the venom of adult specimens of *B. asper*, whereas venoms of neonates have a predominance of P-III SVMP [113]. In *B. jararacussu* venom, P-III-class SVMP with pro-coagulant and hemorrhagic functions were the most abundant components in venom of smaller snakes, while the basic myotoxic PLA_2_ were the major components in larger snakes venoms [99]. Higher hematotoxicity confers more efficient predatory function in the venom from small snakes [112]. These proteomic differences have clinical consequences. Envenomings inflicted by adult *B. atrox* snakes cause more severe local inflammatory effects, whereas venom-induced coagulopathy is more frequent in envenomings caused by juvenile specimens [114]. Similarly, for an equal mass of venom from a small snake than a large one, more antivenom seems to be needed to correct coagulopathy [110].

Few studies reported modifications of the venom in snakes kept in captivity. The venom from *B. atrox* showed a weak variability restricted to the less abundant components in the majority of snakes. However, in some individuals, SVMPs were drastically affected showing the plasticity of the venom phenotype during the lifespan of snakes held in captivity for more than one year [115].

Intraspecies variation in snake venom has consequences on antivenom treatment because snakes of different sex, age or localities may be result in envenomation needing higher doses to achieve total neutralization of lethality or other toxic activities, in particular hemostatic ones [110,116,117]. Variation in the venom of snakes held in captivity raises the question of the effectiveness of antivenoms manufactured from these venoms [76]. However, it is likely that (a) pooling of venoms limits the spectrum of variability and (b) gene and demographic differences are more marked than variations in venoms occurring in captivity [107].

## 4. Pathophysiology of Bleeding

The main classes of toxins isolated from *Bothrops* venoms are endowed with multiple biological activities targeted against hemostasis (Table 2). A multimodal approach combining clinical, animal model-based and in vitro studies has allowed to understand the pathophysiology of bleeding and the respective role of each protein family involved.

### 4.1. Vascular Damage

In patients envenomed by *Bothrops*, systemic bleeding can occur even with clottable blood and normal platelet counts, suggesting than venom-induced vascular damage is primarily responsible for the bleeding [29,45].

In an intravital microscopic observation of the hemorrhage process induced by *B. asper* venom on the mouse cremaster muscle, bleeding started from the capillary segment of the microvasculature, about 5 min after venom exposure. It was explosive, appearing as rapid bursts of erythrocytes into the extravascular space [118]. Similar results were observed with *B. jararaca* venom [119].

Hemorrhage happened *per rhexis* (erythrocytes escape through gaps in damaged endothelial cells) and not *per diapedesis* (through widened intercellular junctions) [120]. In mice, the intramuscularly injection of whole *B. asper* venom induced endothelial cell degeneration in 1 min. On electron microscopy, the morphological pattern of vascular damage associated a reduction in the thickness of endothelial cells with blebs and cytoplasmic projections protruding to the vascular lumen, a decrease in the number of pinocytotic vesicles and a detachment of some endothelial cells from their surrounding basal lamina, leading to gaps in the continuity of endothelial cells then extravasation. No alterations in the intercellular junctions were observed in damaged capillaries [120]. 

All *Bothrops* venoms including *B. lanceolatus* and *B. caribbaeus* ones have hemorrhagic activity (Table 3).

#### 4.1.1. Degradation of Basement Membrane by SVMP

Serum SVMP levels are significantly higher in patients with systemic bleeding compared to those without bleeding in *B. jararaca* envenomation [52]. In experimental murine model, acute vascular damaging effects observed with *B. asper* whole venom were also induced by BaP1 (P-I SVMP) and BaH-1 (P-III SVMP) from *B. asper* venom, suggesting that SVMP have an important role in bleeding [124,125]. When the peptidomimetic matrix metalloproteinase inhibitor batimastat and the chelating agent CaNa_2_EDTA were administered at various time intervals after experimental envenoming in mice at the same site of *B. asper* venom injection, both compounds effectively neutralized local hemorrhage [126]. This highlights the importance of the metalloproteinase-related catalytic activity as major mechanism involved in bleeding.

The ability of SVMP able to induce hemorrhage, named hemorrhagins, is not related to a direct cytotoxic effect on capillary endothelium and the rapid degenerative changes of endothelial cells observed in vivo are the result of the proteolytic degradation of basement membrane (BM) components of microvessels [124,127]. SVMP are able to degrade diverse components such as types I and IV collagen, fibronectin and laminin once incubated with these extracellular matrix proteins in vitro [124,128]. On human umbilical-vein endothelial cell culture, jararhagin decreased endothelial cell viability in a concentration-dependent manner and induced cellular apoptosis, probably due to a catalytic activity suggestive of anoikis, by loss of contact with their matrix [85,129]. Degradation of BM and its related extracellular matrix components by P-III SVMP and P-I SVMP was confirmed in vivo [130,131,132]. SVMP involved in disruption of BM also play an important role in the development of coagulopathy following the rapid venom coagulation component spread from the injected area into the systemic circulation [133].

The proteomic analysis of exudates collected in the vicinity of damaged gastrocnemius muscle of mice showed the early presence of BM components and other extracellular matrix proteins reflecting the rapid microvascular damage induced by SVMP. Presence of fragments of type IV collagen and perlecan one hour after envenoming suggests that hydrolysis of these BM components plays a key role in the genesis of hemorrhage [134]. When comparing the action of P-I hemorrhagic and non-hemorrhagic SVMP (respectively BaP1 and leuc-a from *B. leucurus* venom), a striking difference was noticed regarding the hydrolysis of type IV collagen [132], suggesting the prominent role that this collagen plays in the mechanical stability of BM and the capillary wall. In vivo studies indicated that hydrolysis of collagen IV by mainly P-II and P-III SVMP is crucial in destabilizing microvessel structures and causing hemorrhage [135].

P-II and P-III SVMP bind more specifically to capillary BM than P-I SVMP, owing to the presence of exosites in the disintegrin, disintegrin-like and cysteine-rich domains of these SVMP, thus explaining their generally higher hemorrhagic activity [135,136]. Collagen binding through a motif located in the disintegrin subdomain allows accumulation of jararhagin P-III SVMP at the site of injection, close to capillary vessels, where its catalytic activity leads to a local hemorrhage [137].

However, the presence of non-catalytic domains in the SVMP is not essential for hemorrhagic activity. Atroxlysin-Ia, a P-I SVMP from *B. atrox* venom, has a high hydrolytic activity towards extracellular matrix proteins, including laminin and collagen IV and is able to cause a fast disruption of capillary vessels allowing the induction of hemorrhage similar to those induced by Batroxrhagin, a P-III SVMP isolated from the venom of the same species [138]. 

PLA_2_ may participate in bleeding, by increasing the activity of SVMP. For example, a PLA_2_ from *B. alternatus* venom exhibits no toxicity towards endothelial cells in culture but was found to significantly enhance the detaching activity of balteragin, a hemorrhagic P-III SVMP from the same venom, on endothelial cells [139].

#### 4.1.2. Consequences on the Endothelium

In vitro BM proteins hydrolysis occurred at relatively late time intervals, whereas in vivo hemorrhage developed within minutes of injection [27]. This difference was explained by a “two-step” mechanism. First, hemorrhagic SVMP hydrolyze capillary BM proteins, then hydrostatic pressure and tangential shear stress induce the distention of the capillary wall, resulting in the disruption of endothelial cell integrity and the consequent hemorrhage [140]. Moreover, in experimental conditions, when blood flow was interrupted, no endothelial cell pathology was observed, suggesting that blood flow is essential for a rapid *Bothrops* envenoming-related endotheliopathy [140].

Despite the absence of direct toxicity, *Bothrops* venoms induce endothelial injury which may participate to bleeding. Important increase in plasma soluble thrombomodulin level was reported indicating severe endothelial injury after the intravenous injection of *B. jararaca* venom in rabbits [141]. High levels of soluble thrombomodulin in plasma may favor hemorrhage, due its cofactor activity in the thrombin-mediated activation of protein C and initiation of a profibrinolytic way [142]. Elevation of soluble thrombomodulin due to the crude venom was totally suppressed by pretreatment with heparin, suggesting that endothelial cell injury caused by *B. jararaca* venom is not due to the direct toxicity of SVMP but to thrombin activated by venom [141].

Hemorrhagic Factor 3 (HF3), a hemorrhagic P-III class SVMP from *B. jararaca* venom, cleaves in vivo endothelial glycocalyx proteoglycans such as biglycan, decorin, glypican-1, lumican and syndecan-1, participating to the disruption of microvasculature integrity and contributing to hemorrhage [143].

### 4.2. Platelet Impairments

#### 4.2.1. Thrombocytopenia

In mice, a dose-dependent drop in the number of platelets was observed after the intravenous injection of *B. asper* venom [144]. *B. jararaca* venom directly aggregated washed human and mouse platelets in vitro and stimulated secretion of adenosine triphosphate (ATP), platelet factor 4 (PF4) and β-hexosaminidase, which are present in dense granules, alpha granules and lysosomes of platelets, respectively [145]. 

Thrombocytopenia may be due to different groups of toxins from *Bothrops* venoms. Clinical studies establishing an inverse correlation between platelet count and hemorrhagin level suggested a link between thrombocytopenia and SVMP activity [52]. Hemorrhagic P-III SVMP was shown to contribute to thrombocytopenia since intravenous injection of jararhagin reduced platelet count in a mouse model of *Bothrops* envenomation [144]. Reduction of platelet number was explained by their activation, aggregation and sequestration in response to the extensive blood vessel damage and the intravascular thrombin generation due to SVMP [144]. Moojenactivase, a P-III SVMP from *B. moojeni* venom, induced direct and thrombin-mediated platelet aggregation [146]. However, thrombocytopenia was under the influence of mechanisms not depending on intravascular generation of thrombin such as observed with SVMP [147]. 

Thrombocytin and Platelet-aggregating proteinase (PA-BJ), SVSP isolated from the venom of *B. atrox* and *B. jararaca* respectively, induce platelet aggregation and granule secretion without clotting fibrinogen. Both enzymes induced calcium mobilization in platelets, and cleaved the protease-activated receptor (PAR)1-receptor [148]. Bothrombin from *B. jararaca* venom activate platelets by interacting with GPIb which is a thrombin receptor [149].

Mild thrombocytopenia was observed in patients with either incoagulable or coagulable blood, suggesting an independent mechanism from procoagulant toxins [45]. Bothrocetin from *B. jararaca* venom and Aspercetin from *B. asper* venom induced platelet aggregation in the presence of vWF, promoting its interaction with platelet receptor GPIb and leading to thrombocytopenia and prolonging the bleeding time in mice [150,151]. These C-type lectin-like proteins, by reducing platelet numbers, promoted hemorrhagic lesions initiated by SVMP [150]. 

*Bothrops* PLA_2_ are able to induce platelet aggregation such as bothropstoxin-II, an Asp49 PLA_2_ isolated from *B. jararacussu* venom [152]. The ability of PLA_2_ to induce platelet activation is related to the capacity to hydrolyse phophatidylcholine and the liberation of arachidonic acid [153]. BmooLAAO-I, a LAAO from *B. moojeni* venom induces platelet aggregation although the exact underlying mechanisms are not well understood [154].

#### 4.2.2. Platelet Hypoaggregation

Besides thrombocytopenia, *Bothrops* venom may induce qualitative thrombopathy. In a work studying platelet function during human envenomations by *B. jararaca*, most of patients who presented systemic bleeding showed hypoaggregation to ADP, ristocetin and collagen [46]. 

Basparin A, a prothrombin activator P-III SVMP from *B. asper* venom and jararacussin-I, a TLE SVSP from *B. jararacussu* venom, induced hypoaggregation to ADP in a mouse model. It was suggested that the hypoaggregating effect depends on defibrin(ogen)ation, because fibrinogen is needed for interaction with integrin αIIbβ3 or because the fibrin(ogen) degradation products generated interact and block this integrin receptor [144]. A phosphodiesterase from *B. jararaca* venom, NPP-BJ, is a homodimeric glycoprotein inhibiting platelet aggregation induced by ADP, by hydrolysis of adenylated nucleotides secreted from platelet dense bodies during platelet activation. Polyclonal antibodies raised against this phosphodiesterase could not abolish the lethal activity of *B. jararaca* venom, suggesting a minor contribution to the lethality [155].

The inhibition of ristocetin-induced aggregation has been observed with jararhagin and was attributed to a direct effect on vWF rather than to its action on the GP Ib-IX-V receptor. Jararhagin degrades vWF, in particular the portion of the molecule, which contains the ligand site for the GPIb receptor, the A1 domain [156]. Mutimeric analysis of plasma incubated with *B. jararaca* venom in vitro shown a marked degradation of vWF with loss of high and intermediate molecular weight bands and an increase in low molecular weight fragments, but these results were much less obvious in patients envenomed by this species [45].

In contrast, collagen-induced platelet aggregation is inhibited by jararhagin following its binding to the α_2_-subunit I domain of the platelet surface α_2_β_1_ integrin (collagen receptor) [157]. Jararhagin also cleaves the β_1_ subunit of the same integrin [156]. This inhibition was initially supposed to be linked to the interaction of sequences present in Dis-like and Cys-rich domains of P-III SVM with integrin α_2_β_1_, because this effect is not related to P-I SVMP which lack such sequences [64]. Basparin A also inhibits collagen-dependent platelet aggregation in vitro [158]. In mice, this effect is abolished by batimastat, indicating its dependence on the enzymatic activity and not on the effect of the disintegrin-like and cysteine-rich domains of this protein [144,159].

Some PLA_2_ inhibit platelet aggregation such as BJ- PLA_2_ from *B. jararaca* [160], BthA-I- PLA_2_ from *B. jararacussu* [161], BmooTX-I [162] and Bmoo PLA_2_ [163] from *B. moojeni* and BE-I- PLA_2_ from *B. erythromelas* [164]. The inhibition of platelet aggregation by PLA_2_ is due to the cleavage of the by-products of arachidonic acid [91].

Bl-LAAO from *B. leucurus* venom dose-dependently inhibited platelet aggregation of both human platelet-rich plasma (PRP) and washed platelets [165].

### 4.3. Venom-Induced Consumption Coagulopathy

Crude venoms exhibit strong procoagulant effects in dose-dependent manner in vitro [110,117,166,167]. Study of *Bothrops* venoms by thromboelastographic method in human plasma showed a predominantly procoagulant activity with an increase in coagulation speed and of clot growth velocity [168]. This pathological activation of coagulation leads to incoagulability and defibrin(ogen)ating in vivo by consuming the limited amounts of clotting factors physiologically available following their continuous activation and/or degradation [144,158]. 

#### 4.3.1. Coagulation Factor Activators

SVMP are able to clot citrated plasma by activating prothrombin and/or factor X. P-I SVMP (like bothrojaractivase from *B. jararaca* venom) and P-III SVMP (like basparin A) activate prothrombin to α-thrombin by producing meizothrombin without requiring additional cofactors such as calcium or phospholipids, which ranks them in the group A of snake venom prothrombin activators [158,169,170]. Prothrombin activators found in species other than *Bothrops* spp. are distinguishable from group B (SVMP with a calcium-dependent activity), group C (SVSP similar to factor Xa-factor/Va complex) and group D (SVSP similar to factor Xa). Group B prothrombin activators convert prothrombin to meizothrombin while groups C and D prothrombin activators convert prothrombin to mature thrombin [171]. In addition to cleaving the heavy chain of factor X that results in its activation like with its physiological activators, TF-VIIa and VIIIa-IXa, factor X alpha activators 1 and 2 from *B. atrox* venom produce two other cleavages, i.e., one near the N-terminal end of the heavy chain of factor X, generating factor Xmu and a second located at one extremity of the heavy chain of factor Xa alpha, generating factor Xav [172]. The SVMP moojenactivase induces human plasma clotting in vitro by activating coagulation factors II (prothrombin) and X, which in turn generate α -thrombin and factor Xa, respectively [146].

Thrombocytin, a SVSP isolated from *B. atrox* venom, activates factors V, VIII and XIII [173,174]. Activation of factor V by thrombocytin proceeds via the cleavage of two peptide bonds yielding a product similar to thrombin-activated factor V [174]. 

Activation of these factors by venom leads to generate endogenous thrombin in vitro, even without the addition of TF as a trigger [175]. Thus snakebite-related coagulopathy should be distinct from the usual disseminated intravascular coagulopathy due to a thrombin generation mediated by the TF/VIIa pathway [176].

#### 4.3.2. Thrombine-Like Enzymes

Defibrination also depends on the action of thrombine-like enzymes which directly hydrolyze fibrinogen in fibrin and induce in vitro clotting of fibrinogen solution. Despite their name, TLE show less than 40% similarity with human thrombin [177]. They preferentially release either fibrinopeptide A or B, rarely both with equal efficiency, unlike thrombin. Moreover, TLE do not activate other coagulation factors, especially factor XIII, leading to friable clots without crosslinking of fibrin, readily degraded by plasmin. Finally TLE are not inhibited by thrombin inhibitors such as antithrombin, heparin and hirudin [178]. 

BjussuSP-I, from *B. jararacussu*, induce defibrin(ogen)ation if intravenously or intramuscularly administered, with reduction in plasma fibrinogen concentration. When injected with SVMP BaP1, BjussuSP-I induced a slightly larger hemorrhagic lesion in the skin of mice, suggesting local hemorrhagic activity exacerbation induced by hemorrhagic SVMP [179]. Experiments performed with inhibitors of metalloproteinases and serine proteinases clearly evidenced that SVMP are by far the most important in vitro procoagulant and in vivo defibrin(ogen)ating components in *Bothrops* venom, whereas the TLE play a minor role, probably due to the low content of the latter enzyme in these venoms [64,113,144,147,180].

#### 4.3.3. Anticoagulant Toxins

Bothrojaracin I, a CTL purified from *B. jararaca* venom, inhibits α-thrombin by the non-covalent binding to exosites 1 and 2, decreases the binding of α-thrombin to fibrinogen and thrombomodulin and reduces protein C and prothrombin activation [181,182].

An anticoagulant C-type lectin isolated in *B. jararaca* venom is able to inhibit factors IX and X and protein S in a calcium-dependent fashion [183].

### 4.4. Fibrinogenolysis and Fibrinolysis

#### 4.4.1. Primary Fibrin(ogen)olysis

Non-coagulant proteinases having fibrin(ogen)olytic activity may contribute to fibrinogen consumption without conversion to fibrin. P-I SVMP have direct fibrin(ogen)olytic activity. Their main biological substrate is fibrin(ogen), whose Aα-chain is degraded rapidly and independently of activation of plasminogen. They are termed as α-fibrinogenases, but the other chains of fibrinogen can be substantially degraded during time [79]. For example, Batx-I, from Colombian *B. atrox* venom, is a P-I SVMP degrading preferentially the Aα- and Bβ-chains, but also inducing a partial degradation of the γ-chain [184]. 

Hemorrhagic P-III SVMP may take part to the consumption of clotting factors. Jararaghin cleaves fibrinogen in the C-terminal part of the Aα chains, resulting in the removal of a 23 kDa fragment while β and γ chains remain unaffected. The remaining purified fibrinogen molecule is still fully functional in both platelet aggregation responses to ADP and in its ability to clot plasma by activating thrombin. The only consequence of the α-fibrinogenase activity of jararhagin was abnormal fibrin polymerization by thrombin [185]. In vitro, purified jararhagin induces strong fibrinolytic activity in human and different animal plasmas [186].

SVSP can exhibit fibrin(ogen)olytic without thrombine-like activity. For example, BjSP from *B. jararaca* venom cleave the Aα- and Bβ-chains of fibrinogen without forming fibrin clots [187]. BjussuSP-I, a TLE SVSP from *B. jararacussu* venom, has a primary fibrinolytic activity [188].

#### 4.4.2. Secondary Fibrinolysis

Hyperfibrinolysis can also contribute to fibrinogen and fibrin consumption [48]. In mice, the intravenous injection of SVMP (basparin A) or SVSP (BjussuSP-I and jararacussin-I) induced secondary fibrinolysis, marked by the post-injection increase in fibrin degradation products and D-dimers [144,158,179]. 

In guinea-pig, plasma, purified jararhagin increases tPA activity by causing dissociation of the tPA/PAI-1 complex and promotes fibrinolysis by the inactivation of α_2_-AP [186]. Reptilase, a TLE from *B. jararaca* venom, inactivates two fibrinolysis inhibitors, PAI-1 and α_2_-AP, and thus induces fibrinolysis pathway [189].

## 5. Pathophysiology of Thrombosis

### 5.1. Prothrombotic State Induced by Bothrops Venoms

As stated previously, toxins responsible for platelet aggregation and coagulation activation lead to platelet and coagulation factor consumption, thus promoting bleeding. However, this initial thrombogenic state may cause microthrombi. An in vivo study of local tissue damage after the topical administration of *B. asper* venom on the mouse cremaster muscle showed the formation of thrombi and emboli in veinules, not in arterioles, before the onset of bleedings [118]. Similar results were reported with *B. jararaca* venom [119]. After the intramuscularly injection of *B. asper* venom in mice, numerous capillaries were damaged with platelet aggregates and fibrin deposition in their lumen [120]. The intramuscular injection of *B. jararacussu* and *B. insularis* venoms induced vascular occlusive thrombosis besides hemorrhage, suggesting that ischemia contributed to muscle necrosis [190,191]. In one rat model, the intravenous administration of *B. jararaca* venom combined with stasis of inferior vena cava resulted in venous thrombosis [192,193] while, in another rat model with venom-induced AKI, its administration resulted in intravascular hemolysis and massive fibrin deposition in glomerular capillaries [194]. Based on all these findings, this prothrombotic state appears in relation to multiple factors such as phenotypic switch of endothelial cells, platelet aggregates and hypercoagulability.

#### 5.1.1. TF Surexpression and Endotheliopathy

The intravenous or subcutaneous injection of *B. jararaca* venom in rats increased the activity and expression of TF at the site of venom injection as well as in plasma and lungs [180]. Berythractivase, a P-III SVMP isolated from *B. erythromelas* venom, was demonstrated in vitro to render endothelial cells highly thrombogenic, with releasing of vWF and expression of TF, ICAM-1 and E-selectin [195,196]. Endothelial biomarkers, such as VCAM-1 and angiopoietin-1, may be clinically useful as predictors of *Bothrops* venom-related AKI [197]. Moojenactivase, a P-IIId SVMP owing CTL domain isolated from *B. moojeni* venom, induces a procoagulant behavior in peripheral blood mononuclear cells by increasing TF in vitro [146,198].

#### 5.1.2. Platelet Aggregates and Hypercoagulability

Bothrocetin has been found to induce platelet aggregates and microthrombi in the lung and spleen [199]. Similarly, aspercetin induced the formation of platelet aggregates in the lung microvessels of mice [144]. The intraperitoneal injection of bothrocetin to rats was shown to induce thrombotic thrombocytopenia with vWF depletion The almost total disappearance of intermediate and high molecular weight multimers of vWF, then the recovery of vWF and platelet count was associated with the observation in plasma of unusually large forms of vWF multimers [199]. 

Once bound to fibrin the capacity of batroxobin, a TLE from *B. atrox* venom, to promote fibrin accretion was found 18-fold greater than that of thrombin, that may promote microvascular thrombosis and fibrin deposition [200,201].

In a rat model of *B. jararaca* envenomation, TMA was reported with thrombocytopenia, hemolytic anemia, schizocytes and microcytes [147]. The onset of microangiopathic hemolytic anemia was dependent on thrombin generation induced by SVMP, since pre-treatment of animals with warfarin, an inhibitor of synthesis of vitamin K-dependent coagulation factors, could prevent it. These results indicated that, at least in this species, thrombin generation followed by fibrin formation and deposition in the vascular bed are involved in TMA pathogenesis.

The intravenous injection of moojenactivase induced intravascular hemolysis and red blood cell fragmentation, and microthrombi in small vessels in lungs then hemorrhage in rats. The local ischemia at the injection site seemed due to abundant thrombi formation since this SVMP did not provoke vessel disruption and local hemorrhage [198]. At low doses injected intravenously or intramuscularly to mice, Basparin A only induced defibrin(ogen)ation. At higher doses, its intravenous administration was responsible for numerous occluding thrombi in pulmonary vessels, mainly in arteries and arterioles, leading to rapid death [158]. 

To date, determinants leading to a hemorrhagic or thrombotic phenotype during envenomation remain unknown. Interestingly, patients with TMA did not experience systemic bleeding. Likewise, P-III SVMP such as berythractivase, moojenactivase or basparin A, able of inducing systemic thrombi, are devoid of hemorrhagic activity [129,158,198], suggesting that situations at lower risk of bleeding may favor thrombotic complication onset.

### 5.2. Macrothrombosis during Envenomation by B. lanceolatus and B. caribbaeus

The exact mechanism of macrothrombosis in human envenomation by *B. lanceolatus* and *B. caribbaeus* remains unknown. There is not successful animal model describing the thrombotic manifestation with one of these two species [97,202], suggesting a species-specific effect and making it difficult to understand thrombotic mechanisms. 

#### 5.2.1. Lack of Proaggregating or Procoagulant Activity of *B. lanceolatus* Venom

Both crude *B. caribbaeus* venom and isolated snaclec from these venoms induce platelet aggregation and agglutination in human PRP. The snaclec binds directly to glycoprotein Ib (GPIb) without platelet activation and induces agglutination in washed fixed platelets without requiring vWF, contrary to botrocetin or aspercetin [202]. Despite venom-induced thrombocytopenia in mice, *B. lanceolatus* venom had no direct effect on platelet aggregation in human PRP [97,123]. 

Unlike *B. atrox*, *B. asper* and *B. jararaca*, *B. lanceolatus* and *B. caribbaeus* venoms were not coagulant when tested on human citrated plasma (Table 3), suggesting the absence of prothrombin or factor X activators [97,121,123]. Of note, these studies examined the coagulation of citrated plasma without adding calcium and phospholipids while it has been shown that these co-factors sometimes conditioned the level of procoagulant activity of *Bothrops* toxins [117,166]. *B. lanceolatus* venom dose-dependently clotted purified human fibrinogen, indicating the presence of a thrombine-like enzyme [203] but was devoid of defibrinating activity after intravenous injection in mice [97,121,123]. 

Intravenous injection of *B. caribbaeus* venom induced a degradation of fibrinogen with an increment in FDP levels but without an increase in D-dimer levels, suggesting more a fibrinogenolytic activity without thrombosis in mice than a fibrinolysis shutdown [202]. Thus, to our knowledge, no toxin responsible for platelet aggregation or hypercoagulability has not been identified in the venom of *B. lanceolatus*.

#### 5.2.2. A TMA-Type Mechanism?

The fatal case of *B. lanceolatus* bite reported by Malbranque et al. supported a TMA-type mechanism of injuries [60]. Histopathologic examination showed multi-focal TMA with endothelial-medial dissection by fibrinous thrombi extending from foci of endothelial damage in small arteries and arterioles of brain, heart, lungs, intestine and kidneys. In addition, there was an unusually intense angiogenesis in cerebral infarcts. There was no vasculitis, perivasculitis or perivascular hemorrhage. Occlusive microthrombi were composed of endothelial cellular debris, platelets and fibrin. 

The main TMA-related diseases are thrombotic thrombocytopenic purpura (TTP) and hemolytic uremic syndrome (HUS) [204]. TTP is caused by the increase of highly reactive high molecular weight multimers of vWF caused by deficiency of the specific vWF-cleaving protease ADAMTS13 [205]. Such mechanism is unlikely here as ADAMTS13 activity is most often preserved in patients bitten by *B. lanceolatus* (communication from Dr Joly, French reference laboratory of TMA, Lariboisière Hospital). 

In typical HUS, endothelial activation is induced by a microbial toxin called Shiga toxin, causing a cytotoxic, proapoptotic and prothrombotic phenotype of the endothelial cell, with increased TF expression, vWF release and platelet activation. By contrast, typical HUS is caused by the complement system activation leading to terminal complement complex formation [206].

Venoms of *B. lanceolatus* and *B. caribbaeus* have vascular toxicity. Their intravenous administration induced lung hemorrhage in mice, suggesting the involvement of hemorrhagic SVMP [97,123,202]. Their intramuscular injection in the gastrocnemius muscle induced prominent hemorrhage [97,123]. A hemorrhagic P-I SVMP was isolated in *B. lanceolatus* venom [207] while a P-III SVMP was isolated in *B. caribbaeus* venom [202].

However, the absence of increments in plasma sE-selectin levels suggested the lack of endothelial cell damage after the intravenous *B. caribbaeus* venom injection in mice [202]. Similarly, *B. lanceolatus* venom showed low gelatinolysis activity and probably lacking activity on critical extracellular matrix proteins, which degradation is associated with hemorrhage [98]. *B. lanceolatus* venom showed particularly low toxicity in endothelial cells (EAhy926) compared to *B. jararaca* venom. After 24 h of exposure, *B. lanceolatus* venom appeared about 10 times less toxic than *B. jararaca* venom, while longer times of incubation enhanced *B. lanceolatus* venom cytotoxicity. *B. lanceolatus* venom did not induce adhesion molecules expression such as ICAM-1, VCAM-1 or E-selectin, neither TF expression on vascular endothelial cell membranes [208].

Thus, based on these experimental findings, *B. lanceolatus* venom seems to exhibit poor direct endothelial cell toxicity, suggesting the involvement of an intermediate system able to activate endothelium in vivo. The complement system may induce thrombosis by activating endothelial cells, with the production of C5a and terminal complement complex triggering TF expression [209,210]. *B. lanceolatus* venom can activate all three complement-pathways. In an ex vivo human blood assay, this venom strongly induced the generation of anaphylatoxins, such as C3a, C4a, C5a and the soluble terminal complement complex [211,212]. Venoms from numerous other species from the genus *Bothrops* are able to activate the complement system by one or several activation pathways, generating high quantities of anaphylatoxins by directly cleaving C3 and C5 or by inactivating the regulator C1-INH. These events involved both metalloproteases and serine proteases [213]. 

## 6. Conclusions: Between Scylla and Charybdis

*Bothrops* venoms are endowed with multiple activities on hemostasis, responsible for different clinical manifestations, ranging from local bleeding to thrombosis and/or systemic bleeding. It has been clearly established that bleeding is not due to disseminated intravascular coagulopathy but to the direct multifocal toxicity involving the vessels, platelets and coagulation. Compared to bleeding, microthrombosis is rarely reported in *Bothrops* envenomation. Fibrin deposition induced by venom-related hemostasis disorders seems at least initially not to result in clinical impact. However, the high incidence of further complications such as AKI suggests a larger involvement of hemostasis disorders than initially believed. In some situations, thrombi may lead to TMA. Case reports and experimental studies suggest that thrombosis involvement is more important when hemorrhage is absent and coagulopathy rapidly corrected. This paradoxical observation requires vigilance even in mild-to-moderate envenomation presentations.

Mechanisms responsible for thrombosis during *B. lanceolatus* envenomation are not fully understood. Endothelial damage seems the most likely involved basis. Due to weak venom activity on vessels, the suggested involvement of the complement system based on in vitro studies merits further investigation in vivo. 

The relatively similar composition of the different *Bothrops* venoms suggests that bleeding and thrombosis should not be considered as two different pathologies but rather two sides of the same pathophysiological continuum. Factors predisposing to envenomation with rather hemorrhagic *versus* thrombotic phenotype are unknown. Their identification would allow better understanding hemostasis disorders induced by venoms from a dynamic perspective and thus anticipating their onset when managing a bitten patient.

## Figures and Tables

**Table 1:** Proportions of protein families targeting hemostasis in *Bothrops* venom.

Species	*B. atrox*	*B. asper*	*B. jararaca*	*B. erythromelas*	*B. lanceolatus*	*B. caribbaeus*
Snake venom metalloproteinases (SVMP)	25.8–85.0	30.7–47.4	10.0–64.0	32.5–59.9	42.4–74.2	68.6
PI-SVMP	4.6–65	13.9–35.1	3.6–10.4	2.7–14.4	25.8	30.6
PII-SVMP	4.0–5.2	<0.1–3.9	-	-	-	-
PIII-SVMP	3.1–69	8.2–19.8	6.7–25.2	29.8–45.5	48.4	38.0
Snake venom serine proteases	0.5–21.5	4.4–18.2	3.0–36.0	4.0–9.7	14.4–27.4	4.7
Phospholipases A_2_	4.2–48.0	0.4–45.5	<0.1–20.2	8.1–15.1	4.5–8.6	12.8
L-amino acid oxydases	0.5–16.9	1.1–9.2	<0.1–9.7		2.8–14.0	8.4
Disintegrins	<0.1–3.2	<0.1–7.5	0.2–7	3.4–8.9		1.7
C-type lectin proteins	0.4–13.1	0.3–16.9	9.0–36.0	8.4–21.6	<0.1–4.5	
References	[99,100,101,102,103]	[104,105]	[98,106,107,108]	[109]	[97,98]	[97]

-, Ranges in the medical literature.

**Table 2:** Points of hemostasis targeted by the different families of proteins isolated in *Bothrops* venoms.

Protein Family	Vascular Effect	Platelet Effect	Coagulation Effect	Fibrinolysis Effect
Snake venom metalloproteinases	Hemorrhage	Aggregation Inhibition of aggregation	FII, FX activation Fibrinogen degradation	Fibrin degradation Tissu-type plasminogen activator activation α_2_-antiplasmin inhibition
Snake venom serine proteases		Aggregation	FV, FVIII, FXIII activation Fibrinogen clotting Fibrinogen degradation	Fibrin degradation PAI-1 and α_2_-antiplasmin inhibition
Phospholipases A_2_	Hemorrhage	Aggregation Inhibition of aggregation		
L-amino acid oxydases		Aggregation Inhibition of aggregation		
Disintegrins		Inhibition of aggregation		
C-type lectin proteins		Aggregation Inhibition of aggregation	FIX, FX, FIIa, Protein S inhibition	

**Table 3:** Lethal and hemostatic activities of *Bothrops* venoms.

Species	*B. atrox*	*B. asper*	*B. jararaca*	*B. lanceolatus*	*B. caribbaeus*
Median lethal activity (LD50) (µg/g) ^1^	3.89–4.75 (ip)	3.39–3.79 (ip)	1.89–2.125 (ip)	6 (iv)/12.8 (ip)	3 (iv)/7.5 (ip)
Local minimum hemorrhagic dose (MHD) (µg) ^2^	1.4–2.4	0.8–1.5	0.26	3.6–3.7	0.7
Minimum coagulant concentration (MCC) (µg/mL) ^3^	0.8–3	0.32–1	0.54	NA	NA
Minimum defibrinating dose (MDD) (µg) ^4^	1.7–5	3–5	3.3	NA	NA
References	[121,122]	[121,122]	[122]	[121,123]	[97]

^1^ Venom dose which induces lethality in 50% of mice injected by intraperitoneal (ip) or intravenous (iv) injection. ^2^ Lowest venom dose which induces a hemorrhagic area of 10 mm diameter, 2 h after intradermal injection in mice. ^3^ Lowest venom concentration which induces coagulation of citrated human plasma in 60 s. ^4^ Lowest venom dose, which induces defibrination in mice 1 h after intravenous injection. NA: no activity detected at the highest venom dose tested.
